# Supplementary material for: Newborn care knowledge and practices among care givers of newborns and young infants attending a regional referral hospital in Southwestern Uganda
Source: PLoS One. 2024 May 7;19(5):e0292766. doi: 10.1371/journal.pone.0292766 (PMC11075863; doi:10.1371/journal.pone.0292766)
Supplement: Attachment — Submitted filename: Response to Reviewrs (2).docx [file pone.0292766.s001.docx]

The editor in Chief

PLOS ONE Journal

February 18, 2024

Dear Sir/Madam

**RE: RESPONSE TO THE REVIEWERS**

I have worked on the corrections as advised by the reviewers. Attached are the points raised by the reviewer and the responses to them.

Please address all correspondences about this manuscript to me at [drdorah@yahoo.com](mailto:drdorah@yahoo.com)

PONE-D-23-25688

Newborn care knowledge and practices among care givers of new-born babies attending a regional referral hospital in Southwestern Uganda

1. Please ensure that your manuscript meets PLOS ONE's style requirements, including those for file naming. The PLOS ONE style templates can be found at 
https://journals.plos.org/plosone/s/file?id=wjVg/PLOSOne_formatting_sample_main_body.pdf and 
https://journals.plos.org/plosone/s/file?id=ba62/PLOSOne_formatting_sample_title_authors_affiliations.pdf

***This has been clarified and written to ensure it meets the PLOS ONE’s style requirements***

2. Note from Emily Chenette, Editor in Chief of PLOS ONE, and Iain Hrynaszkiewicz, Director of Open Research Solutions at PLOS: Did you know that depositing data in a repository is associated with up to a 25% citation advantage (https://doi.org/10.1371/journal.pone.0230416)? If you’ve not already done so, consider depositing your raw data in a repository to ensure your work is read, appreciated and cited by the largest possible audience. You’ll also earn an Accessible Data icon on your published paper if you deposit your data in any participating repository (<https://plos.org/open-science/open-data/#accessible-data>).

***This will be de discussed with MUST REC and the other ethical bodies of the university. We have however put an email address where raw data can be accessed on request***

3. Thank you for stating the following in your Competing Interests section:  
"I have read the journal's policy and the authors of this manuscript have no competing interests"

Please complete your Competing Interests on the online submission form to state any Competing Interests. If you have no competing interests, please state "The authors have declared that no competing interests exist.", as detailed online in our guide for authors at http://journals.plos.org/plosone/s/submit-now 
 This information should be included in your cover letter; we will change the online submission form on your behalf.

***This has been stated as required in cover letter***

4. In this instance it seems there may be acceptable restrictions in place that prevent the public sharing of your minimal data. However, in line with our goal of ensuring long-term data availability to all interested researchers, PLOS’ Data Policy states that authors cannot be the sole named individuals responsible for ensuring data access (http://journals.plos.org/plosone/s/data-availability#loc-acceptable-data-sharing-methods).

Data requests to a non-author institutional point of contact, such as a data access or ethics committee, helps guarantee long term stability and availability of data. Providing interested researchers with a durable point of contact ensures data will be accessible even if an author changes email addresses, institutions, or becomes unavailable to answer requests.

Before we proceed with your manuscript, please also provide non-author contact information (phone/email/hyperlink) for a data access committee, ethics committee, or other institutional body to which data requests may be sent. If no institutional body is available to respond to requests for your minimal data, please consider if there any institutional representatives who did not collaborate in the study, and are not listed as authors on the manuscript, who would be able to hold the data and respond to external requests for data access? If so, please provide their contact information (i.e., email address). Please also provide details on how you will ensure persistent or long-term data storage and availability.

***The data that was collected and used for the study will be made available on request from the MUST research Ethics committee of Mbarara University of science and Technology at*** [***uncstresearch@uncst.go.ug***](mailto:uncstresearch@uncst.go.ug) ***and*** [***palele@must.ac.ug***](mailto:palele@must.ac.ug)***. I have stated this in the cover letter***

5. Your ethics statement should only appear in the Methods section of your manuscript. If your ethics statement is written in any section besides the Methods, please delete it from any other section. 

***This has been removed and transferred to the methods section as advised***

Thank you so much

Sincerely,

Dr. Dorah Nampijja

Lecturer, Department of Paediatrics and child Health

Mbarara University of Science and Technology
